# Supplementary figures and images for: The intragenus and interspecies quorum-sensing autoinducers exert distinct control over Vibrio cholerae biofilm formation and dispersal
Source: PLoS Biol. 2019 Nov 11;17(11):e3000429. doi: 10.1371/journal.pbio.3000429 (PMC6872173; doi:10.1371/journal.pbio.3000429)

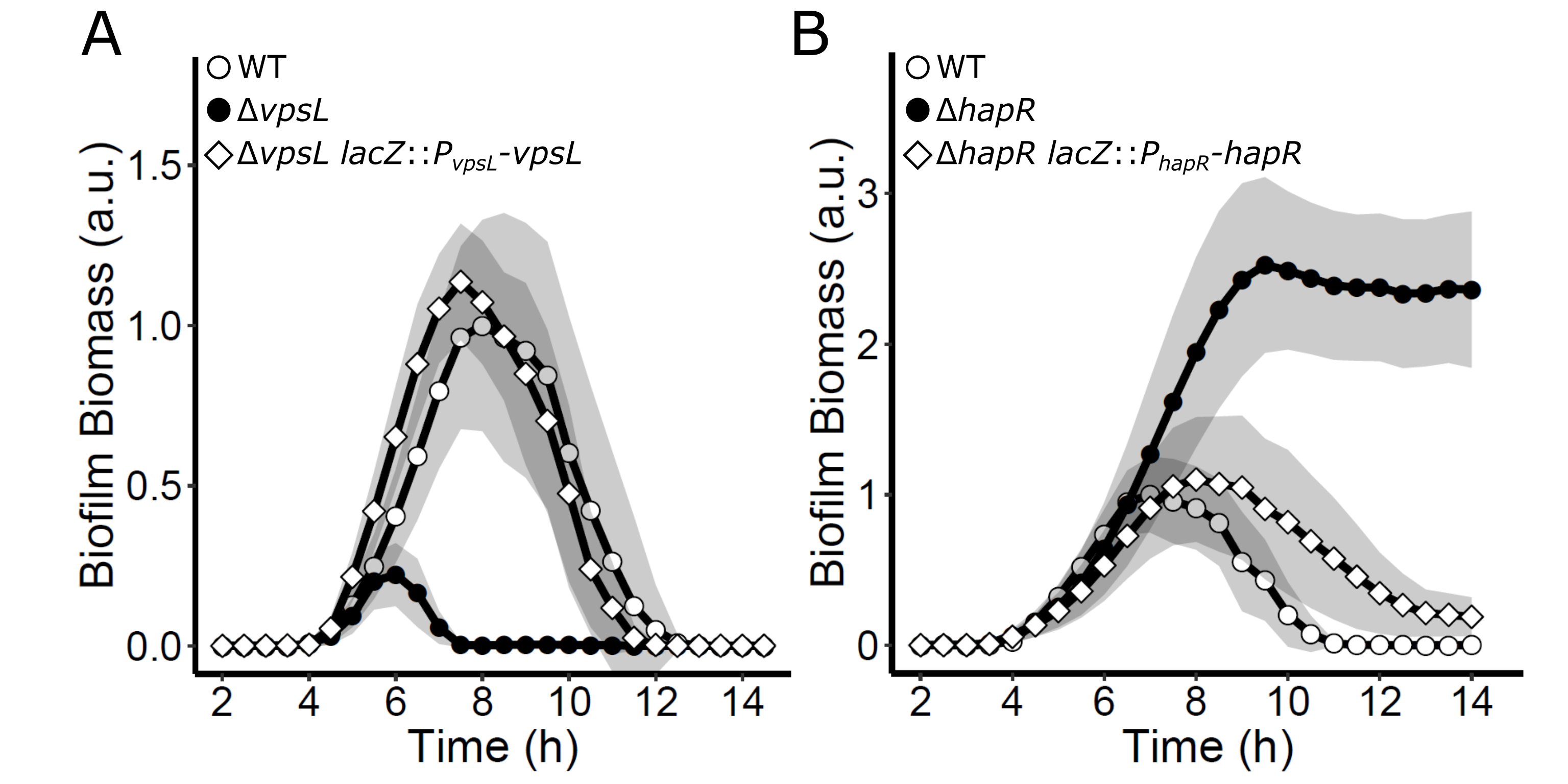

Supplement: S1 Fig — (A) Quantitation of biofilm biomass for V. cholerae WT, the ΔvpsL strain, and the complemented ΔvpsL lacZ::PvpsL-vpsL strain over time. (B) As in A for V. cholerae WT, the ΔhapR strain, and the complemented ΔhapR lacZ::PhapR-hapR strain. Data are represented as means normalized to the peak biofilm biomass of the WT strain in each experiment. In all cases, n = 3 biological and n = 3 technical replicates, ± SD (shaded). Numerical data are available in S1 Data. WT, wild type. (TIF) [file pbio.3000429.s001.tif]

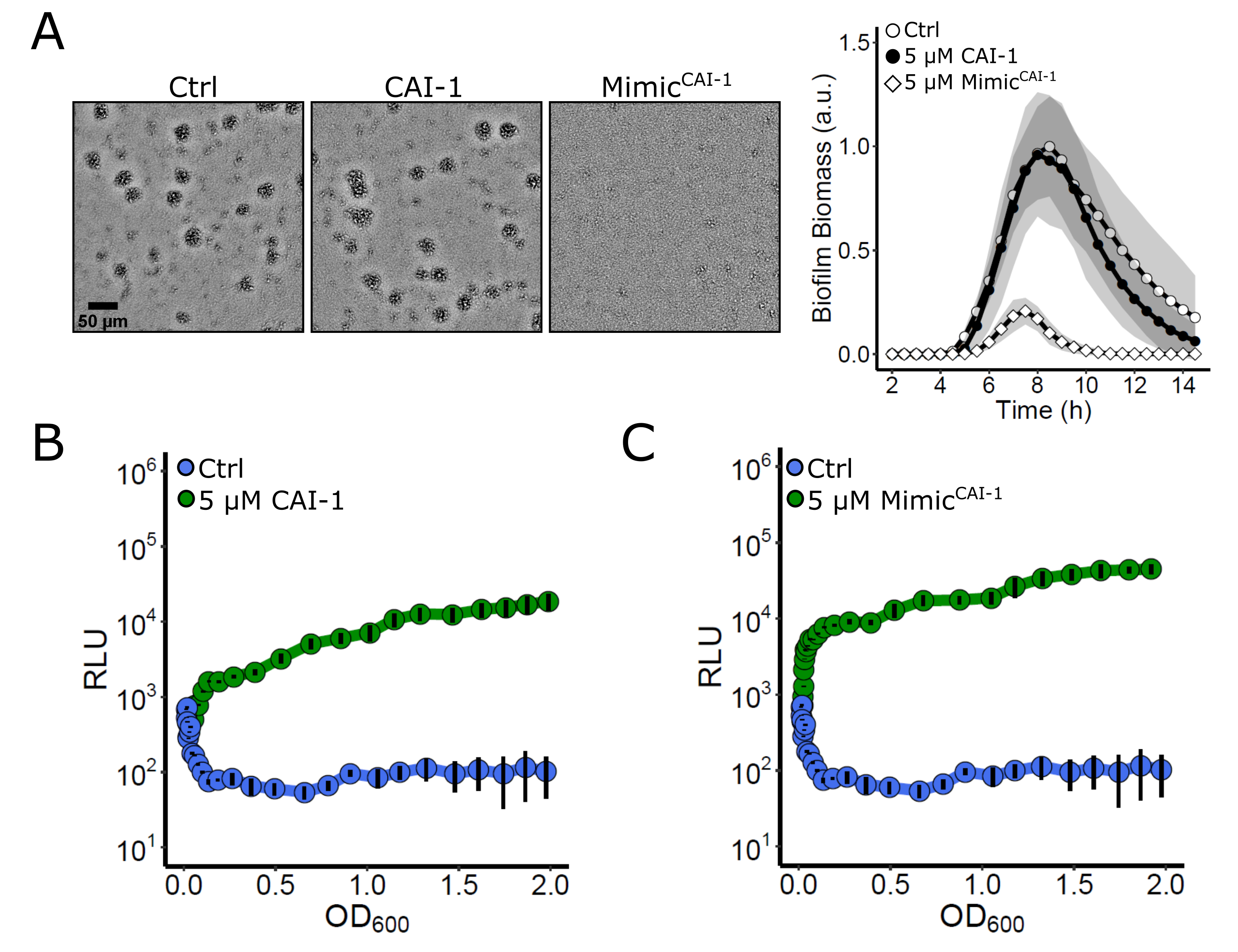

Supplement: S2 Fig — (A) Left panel: representative projections of the V. cholerae CAI-1 reporter strain (ΔvpsS, ΔcqsR, ΔluxQ, ΔcqsA) treated with 0.25% DMSO (Ctrl), 5 μM CAI-1, or 5 μM MimicCAI-1 after 9 h of biofilm growth at 30°C. Right panel: quantitation of biofilm biomass for the strain in A treated with 0.25% DMSO (Ctrl), 5 μM CAI-1, or 5 μM MimicCAI-1 over time. Data are represented as means normalized to the peak biofilm biomass of the DMSO control strain. n = 3 biological and n = 3 technical replicates, ± SD (shaded). (B) The corresponding lux pattern for the strain in A following treatment with 0.25% DMSO (Ctrl) or 5 μM CAI-1. (C) As in B following treatment with 0.25% DMSO (Ctrl) or 5 μM MimicCAI-1. RLUs are defined as light production (a.u.) divided by OD600. For B and C, n = 3 biological replicates and error bars represent SD. Numerical data are available in S1 Data. a.u., arbitrary unit; CAI-1, cholerae autoinducer-1; Ctrl, control; OD, optical density; RLU, relative light unit. (TIF) [file pbio.3000429.s002.tif]

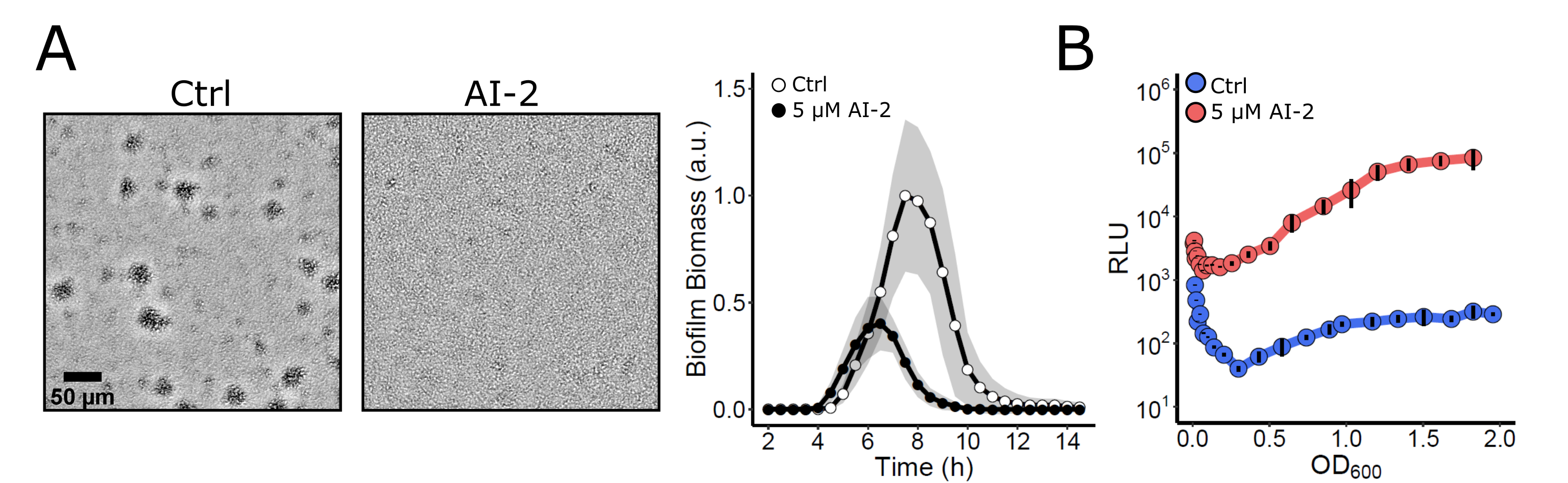

Supplement: S3 Fig — (A) Left panel: representative projections of the V. cholerae AI-2 reporter strain (ΔvpsS, ΔcqsR, ΔcqsS, ΔluxS) treated with 0.25% DMSO (Ctrl) or 5 μM AI-2 after 9 h of biofilm growth at 30°C. Right panel: Quantitation of biofilm biomass for the strain in A treated with 0.25% DMSO (Ctrl) or 5 μM AI-2 over time. Data are represented as means normalized to the peak biofilm biomass of the DMSO control strain. n = 3 biological and n = 3 technical replicates, ± SD (shaded). (B) The corresponding lux pattern for the strain in A following treatment with 0.25% DMSO (Ctrl) or 5 μM AI-2. RLUs are defined as light production (a.u.) divided by OD600. n = 3 biological replicates and error bars represent SD. Numerical data are available in S1 Data. AI-2, autoinducer-2; a.u., arbitrary unit; Ctrl, control; OD, optical density; RLU, relative light unit. (TIF) [file pbio.3000429.s003.tif]

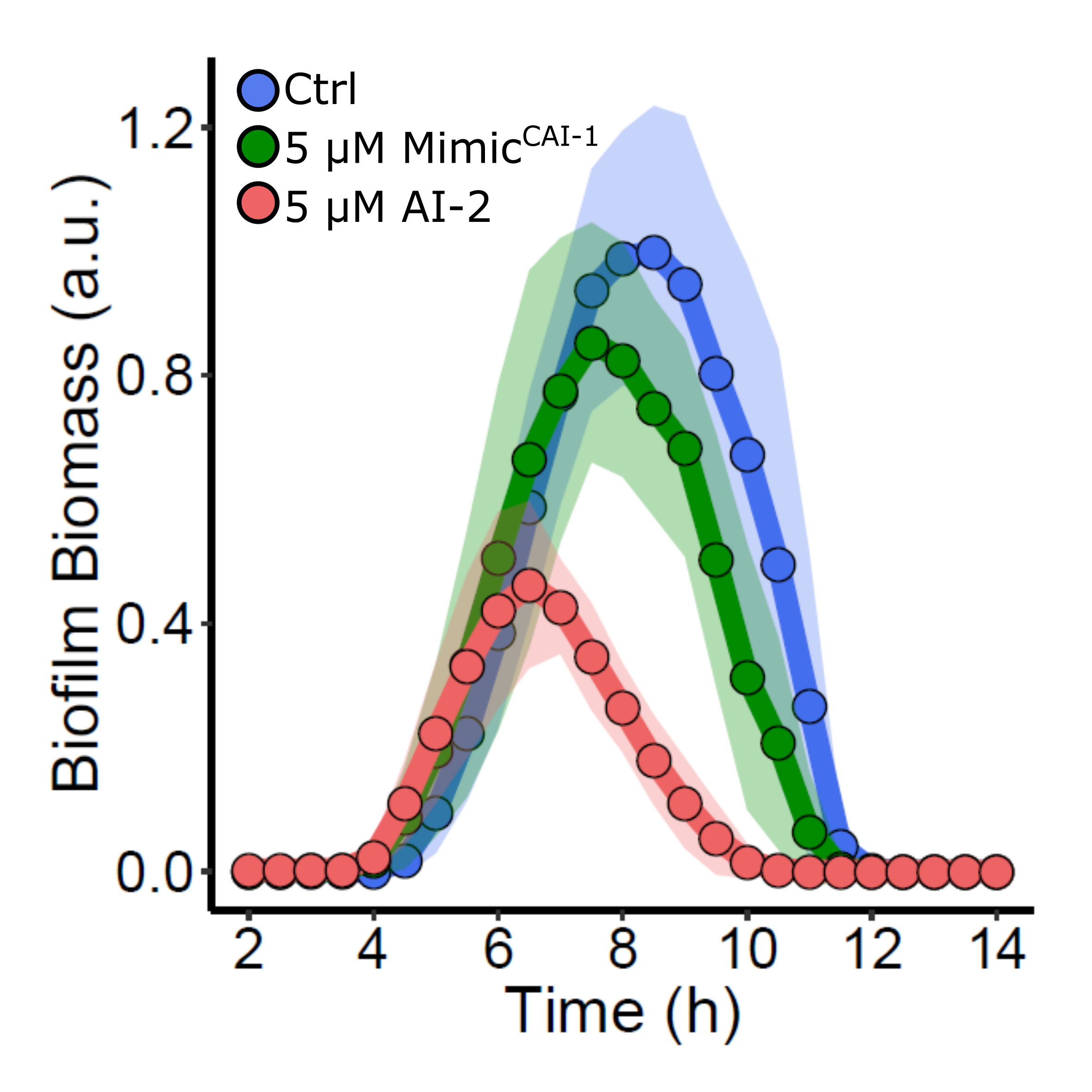

Supplement: S4 Fig — Quantitation of biofilm biomass for the V. cholerae ΔvpsS, ΔcqsR strain treated with 0.25% DMSO (Ctrl), 5 μM MimicCAI-1, or 5 μM AI-2 over time. Data are represented as means normalized to the peak biofilm biomass of the DMSO control strain in each experiment. n = 3 biological and n = 3 technical replicates, ± SD (shaded). Numerical data are available in S1 Data. AI-2, autoinducer-2; Ctrl, control. (TIF) [file pbio.3000429.s004.tif]

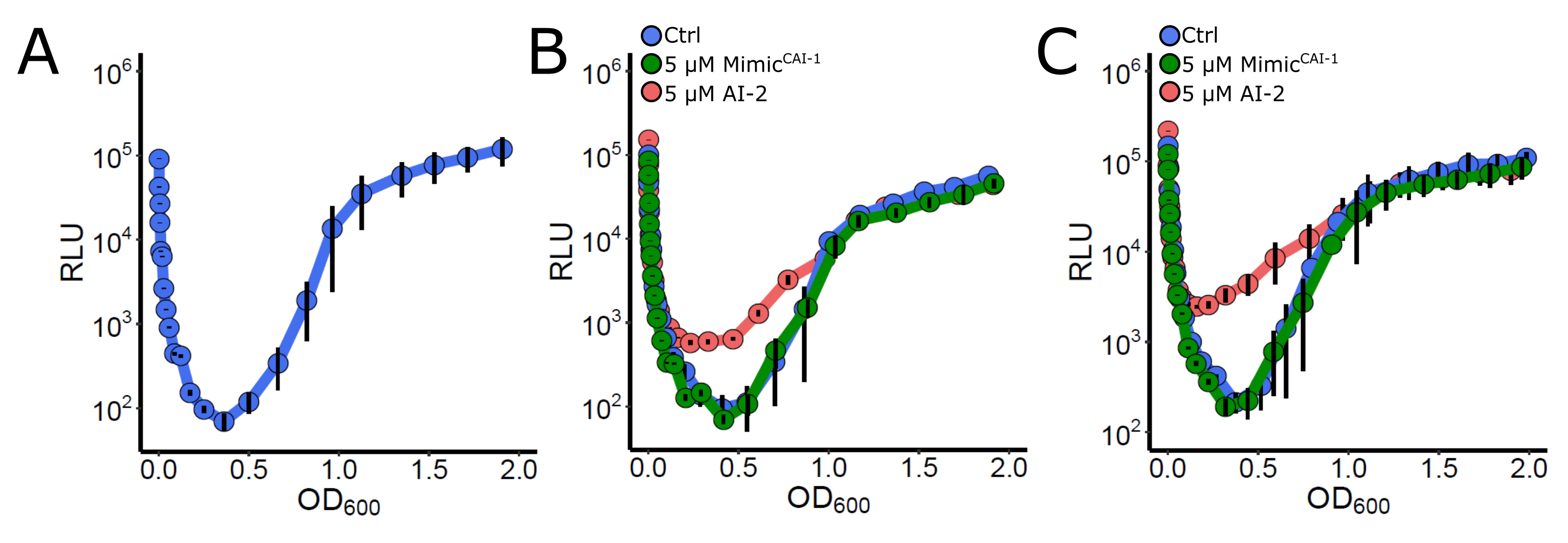

Supplement: S5 Fig — (A) The lux pattern for WT V. cholerae over time. (B) As in A following treatment with 0.25% DMSO (Ctrl), 5 μM MimicCAI-1, or 5 μM AI-2. (C) As in B for the ΔvpsS, ΔcqsR strain. RLUs are defined as light production (a.u.) divided by OD600. n = 3 biological replicates and error bars represent SD. Numerical data are available in S1 Data. AI-2, autoinducer-2; a.u., arbitrary unit; CAI-1, cholerae autoinducer-1; Ctrl, control; OD, optical density; RLU, relative light unit; WT, wild type. (TIF) [file pbio.3000429.s005.tif]

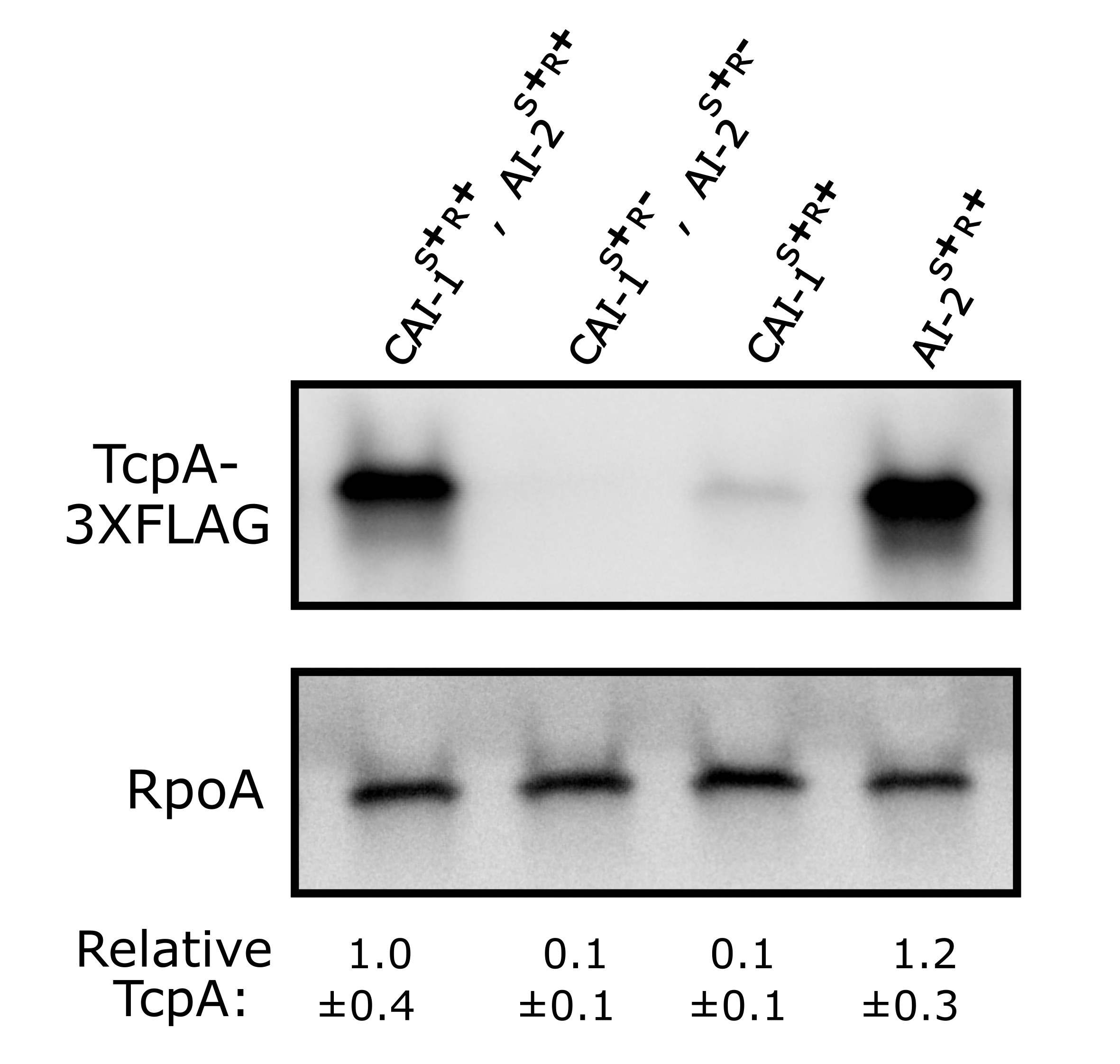

Supplement: S6 Fig — Representative western blot showing TcpA-3×FLAG in the V. cholerae strain possessing both the CqsS and LuxPQ QS circuits (AI-2S+R+, CAI-1S+R+; first lane), lacking all QS receptors (AI-2S+R−, CAI-1S+R−; second lane), possessing only the CAI-1 QS circuit (CAI-1S+R+; third lane), and possessing only the AI-2 QS circuit (AI-2S+R+, fourth lane). RpoA was used as the loading control. Quantification is based on three biological replicates for each condition. Values were normalized to the strain possessing both QS circuits. Numerical data are available in S1 Data. AI-2, autoinducer-2; CAI-1, cholerae autoinducer-1; LCD, low cell density; QS, quorum sensing. (TIF) [file pbio.3000429.s006.tif]

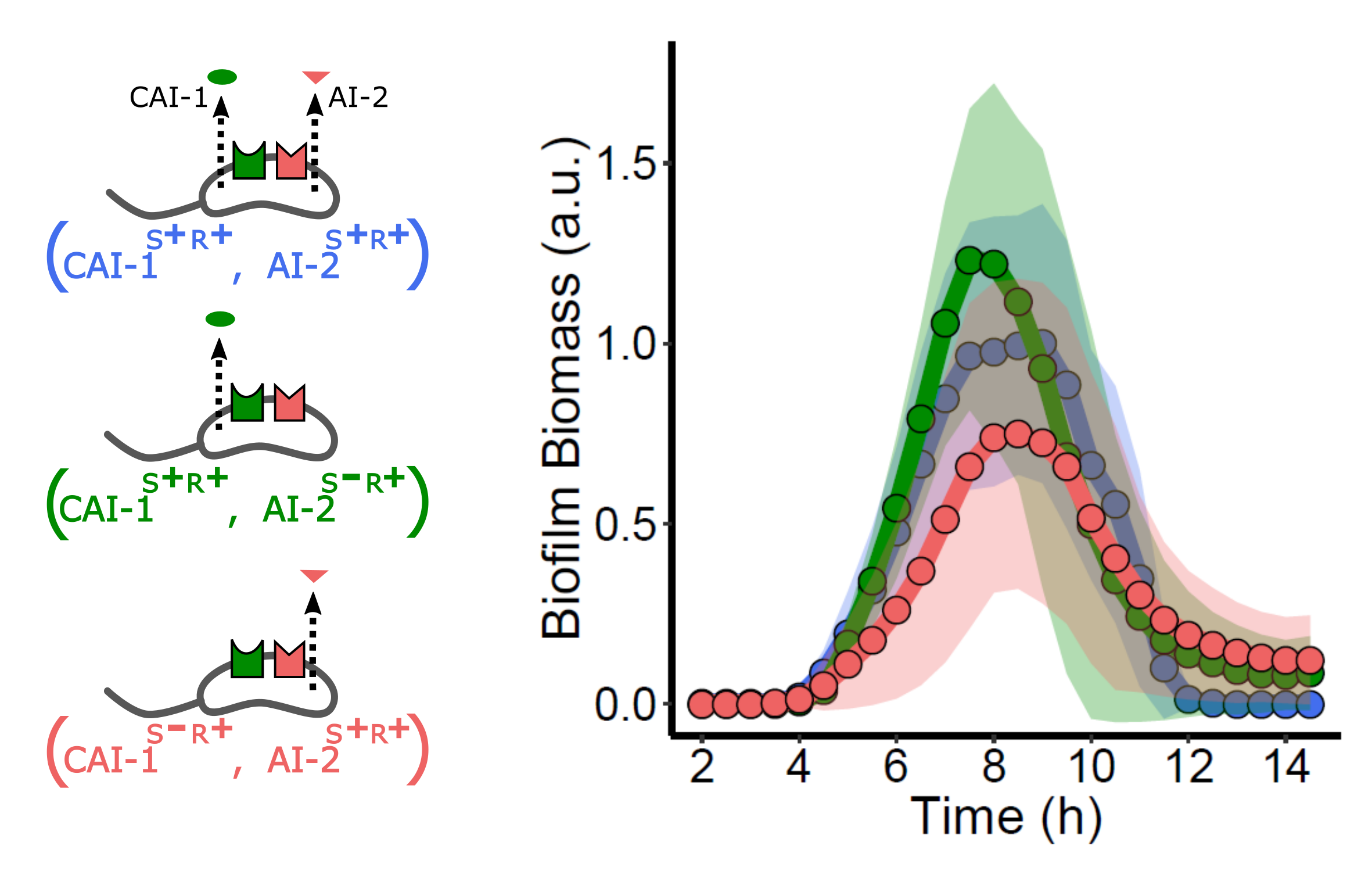

Supplement: S7 Fig — Left panel: schematic representing V. cholerae strains used in the right panel. Right panel: quantitation of biofilm biomass over time for the strain possessing both QS receptors and synthases (AI-2S+R+, CAI-1S+R+; blue), both QS receptors but lacking luxS (AI-2S−R+, CAI-1S+R+; green), and both QS receptors but lacking cqsA (AI-2S+R+, CAI-1S−R+; red). Data are represented as means normalized to the peak biofilm biomass of the WT strain in each experiment. In all cases, n = 3 biological and n = 3 technical replicates, ± SD (shaded). Numerical data are available in S1 Data. AI-2, autoinducer-2; QS, quorum sensing; WT, wild type. (TIF) [file pbio.3000429.s007.tif]
