# Supplementary material for: The intragenus and interspecies quorum-sensing autoinducers exert distinct control over Vibrio cholerae biofilm formation and dispersal
Source: PLoS Biol. 2019 Nov 11;17(11):e3000429. doi: 10.1371/journal.pbio.3000429 (PMC6872173; doi:10.1371/journal.pbio.3000429)
Supplement: S1 Table — (DOCX) [file pbio.3000429.s010.docx]

**S1 Table**

| Strain Number | Genotype | Ab^R*^ | Parent |
| --- | --- | --- | --- |
| AB_Vc_102 | WT O1 El Tor biotype C6706str2 | Sm |  |
| AB_Vc_479 | Δ*vc1807*::Kan^R^ (Referred to as WT) | Sm, Kan | AB_Vc_102 |
| AB_Vc_487 | Δ*vpsL* Δ*vc1807::*Kan^R^ | Sm, Kan | AB_Vc_102 |
| AB_Vc_675 | Δ*vpsL* Δl*acIZ*::*PvpsL-vpsL* Δ*vc1807::*Kan^R^ | Sm, Kan | AB_Vc_102 |
| AB_Vc_481 | *luxOD61A* Δ*vc1807*::Kan^R^ | Sm, Kan | AB_Vc_102 |
| AB_Vc_483 | *luxOD61E* Δ*vc1807::*Kan^R^ | Sm, Kan | AB_Vc_102 |
| AB_Vc_235 | Δ*hapR* Δ*vc1807*::Spec^R^ | Sm, Spec | AB_Vc_102 |
| AB_Vc_684 | Δ*hapR* Δ*lacIZ*::*PhapR-hapR* Δ*vc1807::*Kan^R^ | Sm, Kan | AB_Vc_102 |
| AB_Vc_633 | Δ*vqmR* Δ*vc1807*::Kan^R^ | Sm, Kan | AB_Vc_102 |
| AB_Vc_491 | Δ*vpsS* Δ*cqsR* Δ*vc1807*::Kan^R^ | Sm, Kan | WN_3369 |
| AB_Vc_280 | *aphA-mNeonGreen* Δ*vc1807*::*Ptac-mRuby3*-Spec^R^ | Sm, Spec | AB_Vc_102 |
| AB_Vc_286 | *hapR-mNeonGreen* Δ*vc1807*::*Ptac-mRuby3*-Spec^R^ | Sm, Spec | AB_Vc_102 |
| AB_Vc_660 | *tcpA-3XFLAG* Δ*vc1807::*Kan^R^ | Sm, Kan | AB_Vc_102 |
| AB_Vc_672 | *tcpA-3XFLAG* Δ*vpsS* Δ*cqsR* Δ*vc1807*::Cm^R^ | Sm, Cm | WN_3369 |
| AB_Vc_668 | *tcpA-3XFLAG* Δ*cqsS* Δ*luxQ* Δ*vpsS* Δ*cqsR* Δ*vc1807*::Cm^R^ | Sm, Cm | WN_3354 |
| AB_Vc_670 | *tcpA-3XFLAG* Δ*luxQ* Δ*vpsS* Δ*cqsR* Δ*vc1807*::Cm^R^ | Sm, Cm | WN_3628 |
| AB_Vc_674 | *tcpA-3XFLAG* Δ*cqsS* Δ*vpsS* Δ*cqsR* Δ*vc1807*::Cm^R^ | Sm, Cm | WN_3627 |
| AB_Vc_455 | Δ*luxQ* Δ*vpsS* Δ*cqsR* Δ*vc1807*::Kan^R^ | Sm, Kan | WN_3628 |
| AB_Vc_459 | Δ*cqsS* Δ*vpsS* Δ*cqsR* Δ*vc1807*::Kan^R^ | Sm, Kan | WN_3627 |
| AB_Vc_467 | Δ*luxPQ*::*cqsS* Δ*cqsS* Δ*vpsS* Δ*cqsR* Δ*vc1807*::Kan^R^ | Sm, Kan | WN_3354 |
| AB_Vc_594 | Δ*cqsS*::*luxPQ* Δ*luxPQ* Δ*vpsS* Δ*cqsR* Δ*vc1807*::Kan^R^ | Sm, Kan | WN_3354 |
| AB_Vc_534 | Δ*luxPQ*::*cqsS* Δ*cqsS*::*luxPQ* Δ*vc1807*::Spec^R^ | Sm, Spec | AB_Vc_102 |
| AB_Vc_499 | Δ*luxS* Δ*cqsS* Δ*vpsS* Δ*cqsR* Δ*vc1807*::Kan^R^ | Sm, Kan | WN_3627 |
| AB_Vc_504 | Δ*cqsA* Δ*luxQ* Δ*vpsS* Δ*cqsR* Δ*vc1807*::Kan^R^ | Sm, Kan | WN_3628 |
| AB_Vc_461 | Δ*luxS* Δ*vpsS* Δ*cqsR* Δ*vc1807*::Kan^R^ | Sm, Kan | WN_3369 |
| AB_Vc_501 | Δ*cqsA* Δ*vpsS* Δ*cqsR* Δ*vc1807*::Kan^R^ | Sm, Kan | WN_3369 |
| AB_Vc_598 | Δ*luxPQ*::*cqsS-3XFLAG* Δ*cqsS*::*luxPQ-3XFLAG* Δ*vpsS* Δ*cqsR* Δ*vc1807*::Kan^R^ | Sm, Kan | WN_3369 |
| AB_Vc_596 | *cqsS-3XFLAG luxQ-3XFLAG* Δ*vpsS* Δ*cqsR* Δ*vc1807*::Kan^R^ | Sm, Kan | WN_3369 |
| AB_Vc_517 | *cqsS-3XFLAG luxQ-3XFLAG* Δ*vc1807*::*PluxC-luxCDABE*::Spec^R^ | Sm, Spec | AH_421 |
| AB_Vc_591 | *cqsS-3XFLAG luxQ-3XFLAG* Δ*vpsS* Δ*cqsR* Δ*vc1807*::*PluxC-luxCDABE*::Spec^R^ | Sm, Spec | AH_399 |
| AB_Vc_519 | *luxQ-3XFLAG* Δ*cqsS* Δ*vpsS* Δ*cqsR* Δ*vc1807*::*PluxC-luxCDABE*::Spec^R^ | Sm, Spec | AH_404 |
| AB_Vc_525 | *cqsS-3XFLAG* Δ*luxQ* Δ*vpsS* Δ*cqsR* Δ*vc1807*::*PluxC-luxCDABE*::Spec^R^ | Sm, Spec | AH_399 |
| AB_Vc_521 | *luxQ-3XFLAG* Δ*luxS* Δ*cqsS* Δ*vpsS* Δ*cqsR* Δ*vc1807*::*PluxC-luxCDABE*::Spec^R^ | Sm, Spec | AH_404 |
| AB_Vc_523 | *cqsS-3XFLAG* Δ*cqsA*(TTT->AA- at codon 9) Δ*vpsS* Δ*cqsR* Δ*luxQ* Δ*vc1807*::*PluxC-luxCDABE*::Spec^R^ | Sm, Spec | AH_399 |
| AB_Vc_593 | Δ*cqsS*::*luxPQ-3XFLAG* Δ*luxPQ* Δ*vpsS* Δ*cqsR* Δ*vc1807*::*PluxC-luxCDABE*::Spec^R^ | Sm, Spec | WN_3354 |
| AB_Vc_601 | Δ*luxPQ*::*cqsS-3XFLAG* Δ*cqsS* Δ*vpsS* Δ*cqsR* Δ*vc1807*::*PluxC-luxCDABE*::Spec^R^ | Sm, Spec | WN_3354 |
| AB_Vc_542 | Δ*cqsS* Δ*luxQ* Δ*vpsS* Δ*cqsR* Δ*vc1807*::*PluxC-luxCDABE*::Spec^R^ | Sm, Spec | WN_3354 |
| AB_Vc_621 | *cqsS-3XFLAG* *luxQ-3XFLAG* Δ*cqsA*(TTT->AA- at codon 9) Δ*vpsS* Δ*cqsR* Δ*vc1807*::*PluxC-luxCDABE*::Spec^R^ | Sm, Spec | AH_404 |
| AB_Vc_625 | *cqsS-3XFLAG luxQ-3XFLAG* Δ*luxS* Δ*vpsS* Δ*cqsR* Δ*vc1807*::*PluxC-luxCDABE*::Spec^R^ | Sm, Spec | AH_399 |
| AB_Vc_548 | *hapR-mNeonGreen* Δ*luxQ* Δ*vpsS* Δ*cqsR* Δ*vpsL*::Cm^R^ Δ*vc1807*::*Ptac-mRuby3*::Spec^R^ | Sm, Cm, Spec | WN_3628 |
| AB_Vc_552 | *hapR-mNeonGreen* Δ*cqsS* Δ*vpsS* Δ*cqsR* Δ*vpsL*::Cm^R^ Δ*vc1807*::*Ptac-mRuby3*::Spec^R^ | Sm, Cm, Spec | WN_3627 |
| AB_Vc_556 | *hapR-mNeonGreen* Δ*cqsA* Δ*luxQ* Δ*vpsS* Δ*cqsR* Δ*vpsL*::Cm^R^ Δ*vc1807*::Spec^R^ | Sm, Cm, Spec | AB_Vc_548 |
| AB_Vc_560 | *hapR-mNeonGreen* Δ*luxS* Δ*cqsS* Δ*vpsS* Δ*cqsR* Δ*vpsL*::Cm^R^ Δ*vc1807*::Spec^R^ | Sm, Cm, Spec | AB_Vc_552 |

*Ab^R^ = Antibiotic Resistance
