# Supplementary material for: The intragenus and interspecies quorum-sensing autoinducers exert distinct control over Vibrio cholerae biofilm formation and dispersal
Source: PLoS Biol. 2019 Nov 11;17(11):e3000429. doi: 10.1371/journal.pbio.3000429 (PMC6872173; doi:10.1371/journal.pbio.3000429)
Supplement: S2 Table — (DOCX) [file pbio.3000429.s011.docx]

**S2 Table**

| **Oligo #** | **Name** | **Purpose** | **Direction** | **5' to 3' Sequence** |
| --- | --- | --- | --- | --- |
| 380 | *vpsL*_3000_up | MUGENT | F | GTGTTAAGAGCACCGATTGCACTTGATC |
| 381 | *vpsL*_3000_down | MUGENT | R | CGTCAGGGTCTGGAACTCAGATTTACG |
| 154 | *luxO*_3000_up | MUGENT | F | CCGCTATTGAGCTGTATTCACTTATCCAC |
| 155 | *luxO*_3000_down | MUGENT | R | CGATTGAATGGTCGAGGTGCCAATCTC |
| 113 | *hapR*_3000_up | MUGENT | F | CAGTGGCACATCATCGTCATC |
| 114 | *hapR*_3000_down | MUGENT | R | CACGCTGAACCACACATTGTTC |
| 248 | *cqsS*_3000_up | MUGENT | F | CGATTTGCTACGCCTTGTATGGC |
| 249 | *cqsS*_3000_down | MUGENT | R | GATCGCTAAAATGTGGTTCCCAG |
| 415 | *cqsA*_3000_up | MUGENT | F | CCGAGGTACTGATATGAACGTTTTGATTCC |
| 416 | *cqsA*_3000_down | MUGENT | R | GATGGATGGTTTGCAACGTGTCGC |
| 256 | *luxQ*_3000_up | MUGENT | F | CTTCTCAATACGCTGAACTAGAACAAGAAG |
| 257 | *luxQ*_3000_down | MUGENT | R | CATCATGCTTAATCCGTACCTATCTACTGTTTATG |
| 274 | *luxS*_3000_up | MUGENT | F | CTGCTGCAAGAAGGCAGCCAA |
| 275 | *luxS*_3000_down | MUGENT | R | GGAGCTTAGAGAGTTTGCCTACGGATGT |
| 105 | *vc1807*_3000_up | MUGENT | F | TTTAAAGGGGATCAGTGACCG |
| 106 | *vc1807*_3000_down | MUGENT | R | CAATTTTGCTTTTGGACCATCCC |
| 111 | *aphA*_3000_up | MUGENT | F | GCTGCGCTCAAAAGTAACGTAAG |
| 112 | *aphA*_3000_down | MUGENT | R | CAGGTCAAACCGCACGTGAAAGTG |
| 280 | *lacIZ*_3000_up_F | MUGENT | F | GAATTTGATGGTCTGTTTATTCGCGCC |
| 285 | *lacIZ*_3000_down_R | MUGENT | R | CGATTTGTTGACGAGATCAAACAAG |
| 15 | *aphA*-*mNG*_B | *aphA*-*mNeonGreen*_SOE | R | AGATCCACTACCACTTCCTGAACCTGCCATCGCGTTCAATTCTGCC |
| 78 | *aphA*-*mNG*_C | *aphA*-*mNeonGreen*_SOE | F | CTCCTCGCCCTTGCTCACCATAGATCCACTACCACTTCCTG |
| 71 | *aphA*-*mNG*_D | *aphA*-*mNeonGreen*_SOE | R | TTACTTGTACAGCTCGTCCATGCCCATCAC |
| 79 | *aphA*-*mNG*_E | *aphA*-*mNeonGreen*_SOE | F | CATGGACGAGCTGTACAAGTAAGCCAAGCCAAACCTGTCGATG |
| 121 | *hapR*-*10aa*-*mNG*_B | *hapR*-*mNeonGreen*_SOE | R | TCCTGATCCGCTGCCTGAGCCGCTTCCTGAGTTCTTATAGATACACAGCATATTGAGGTAGCTATC |
| 166 | *hapR*-*10aa*-*mNG*_C | *hapR*-*mNeonGreen*_SOE | F | AGGAAGCGGCTCAGGCAGCGGATCAGGAATGGTGAGCAAGGGCGAGGAGGATAAC |
| 167 | *hapR*-*10aa*-*mNG*_D | *hapR*-*mNeonGreen*_SOE | R | GCGCCCTTTGTGCTGCCCAAGAAATTACTTGTACAGCTCGTCCATGCCCAT |
| 168 | *hapR*-*10aa*-*mNG*_E | *hapR*-*mNeonGreen*_SOE | F | ATGGGCATGGACGAGCTGTACAAGTAATTTCTTGGGCAGCACAAAGGGCGC |
| 296 | Δ*luxPQ*::*cqsS*_B | Δ*luxPQ*::*cqsS*_SOE | R | CTTTATTACATCCATGCTCACTATCACAGCTTCCTCATGAGCTTTTCTTC |
| 297 | Δ*luxPQ*::*cqsS*_C | Δ*luxPQ*::*cqsS*_SOE | F | GAAGAAAAGCTCATGAGGAAGCTGTGATAGTGAGCATGGATGTAATAAAG |
| 298 | Δ*luxPQ*::*cqsS*_D | Δ*luxPQ*::*cqsS*_SOE | R | GTTCTCGAACACGCTTTTCTCGCTGGCCTACACCCAAGCTGCCACTTTATTTAG |
| 299 | Δ*luxPQ*::*cqsS*_E | Δ*luxPQ*::*cqsS*_SOE | F | CTAAATAAAGTGGCAGCTTGGGTGTAGGCCAGCGAGAAAAGCGTGTTCGAGAAC |
| 302 | Δ*cqsS*::*luxPQ*_B | Δ*cqsS*::*luxPQ*_SOE | R | CAGAAACAGCGGAGATATTAGCTTTCTTTTCATTACCGTTGCATTCTCTTGCTAATCATC |
| 303 | Δ*cqsS*::*luxPQ*_C | Δ*cqsS*::*luxPQ*_SOE | F | GATGATTAGCAAGAGAATGCAACGGTAATGAAAAGAAAGCTAATATCTCCGCTGTTTCTG |
| 304 | Δ*cqsS*::*luxPQ*_D | Δ*cqsS*::*luxPQ*_SOE | R | TGCAGCTTCAAGTAGGAAGGGTATAGTCAATTTAAGCCAGCGTTTTTTTGGCC |
| 305 | Δ*cqsS*::*luxPQ*_E | Δ*cqsS*::*luxPQ*_SOE | F | GGCCAAAAAAACGCTGGCTTAAATTGACTATACCCTTCCTACTTGAAGCTGCA |
| 471 | *tcpA_3XFLAG*_B | *tcpA-3XFLAG*_SOE | R | CCCGTCCCTGAAAATACAGGTTTTCACTGTTACCAAAAGCTACTGTGAATGG |
| 472 | *tcpA_3XFLAG*_C | *tcpA-3XFLAG*_SOE | F | CCATTCACAGTAGCTTTTGGTAACAGTGAAAACCTGTATTTTCAGGGACGGG |
| 473 | *tcpA_3XFLAG*_D | *tcpA-3XFLAG*_SOE | R | CTTGTAATAACTCCCAGCAGCGACCAATGCCATCCCTAATAC |
| 474 | *tcpA_3XFLAG*_E | *tcpA-3XFLAG*_SOE | F | GTATTAGGGATGGCATTGGTCGCTGCTGGGAGTTATTACAAG |
| 231 | *Ptac*-*mRuby3*:: Spec^R^ _B | *Ptac-mRuby3*::Spec^R^_SOE | R | CCTTAGCTACCCGCCTTCTGTAC |
| 234 | *Ptac*-*mRuby3*:: Spec^R^ _C | *Ptac-mRuby3*::Spec^R^*_*SOE | F | GTACAGAAGGCGGGTAGCTAAGGTGCACCAATGCTTCTGGCGTCAG |
| 235 | *Ptac*-*mRuby3*:: Spec^R^ _D | *Ptac-mRuby3*::Spec^R^*_*SOE | R | GTCGACGGATCCCCGGAATTTATTACTTATATAATTCATCCATTCCACCC |
| 232 | *Ptac*-*mRuby3*:: Spec^R^ _E | *Ptac-mRuby3*::Spec^R^*_*SOE | F | ATTCCGGGGATCCGTCGAC |
| 328 | *LacIZ*_Universal_B | Δ*lacIZ*::*PvpsL-vpsL*_SOE | R | AAGATTCCTTCTCTATCACAGGCGCAATAG |
| 323 | Δ*lacIZ*::*PvpsL*-vpsL_C | Δ*lacIZ*::*PvpsL-vpsL*_SOE | F | CGCCTGTGATAGAGAAGGAATCTTTTTGATTAACCTATTAACCATCATAAAAG |
| 515 | Δ*lacIZ*::*PvpsL*-vpsL_D | Δ*lacIZ*::*PvpsL-vpsL*_SOE | R | GACTTCTTTACTCCTCGGCTTGAGGGTTAATACGCGTTTTTTCCAACAAATCCTTTG |
| 516 | Δ*lacIZ*::*PvpsL*-vpsL_E | Δ*lacIZ*::*PvpsL-vpsL*_SOE | F | CAAAGGATTTGTTGGAAAAAACGCGTATTAACCCTCAAGCCGAGGAGTAAAGAAGTC |
| 328 | *LacIZ*_Universal_B | Δ*lacIZ*::*PhapR-hapR*_SOE | R | AAGATTCCTTCTCTATCACAGGCGCAATAG |
| 332 | Δ*lacIZ*::*PhapR*-*hapR*_C | Δ*lacIZ*::*PhapR-hapR*_SOE | F | CTATTGCGCCTGTGATAGAGAAGGAATCTTCCATTCTCGTTGTGTTGGGCG |
| 517 | Δ*lacIZ*::*PhapR*-*hapR*_D | Δ*lacIZ*::*PhapR-hapR*_SOE | R | GACTTCTTTACTCCTCGGCTTGAGGGTCAGTTCTTATAGATACACAGCATATTGAGG |
| 518 | Δ*lacIZ*::*PhapR*-*hapR*_E | Δ*lacIZ*::*PhapR-hapR*_SOE | F | CCTCAATATGCTGTGTATCTATAAGAACTGACCCTCAAGCCGAGGAGTAAAGAAGTC |
