# Supplementary material for: The intragenus and interspecies quorum-sensing autoinducers exert distinct control over Vibrio cholerae biofilm formation and dispersal
Source: PLoS Biol. 2019 Nov 11;17(11):e3000429. doi: 10.1371/journal.pbio.3000429 (PMC6872173; doi:10.1371/journal.pbio.3000429)

Gel images for Figure 4F

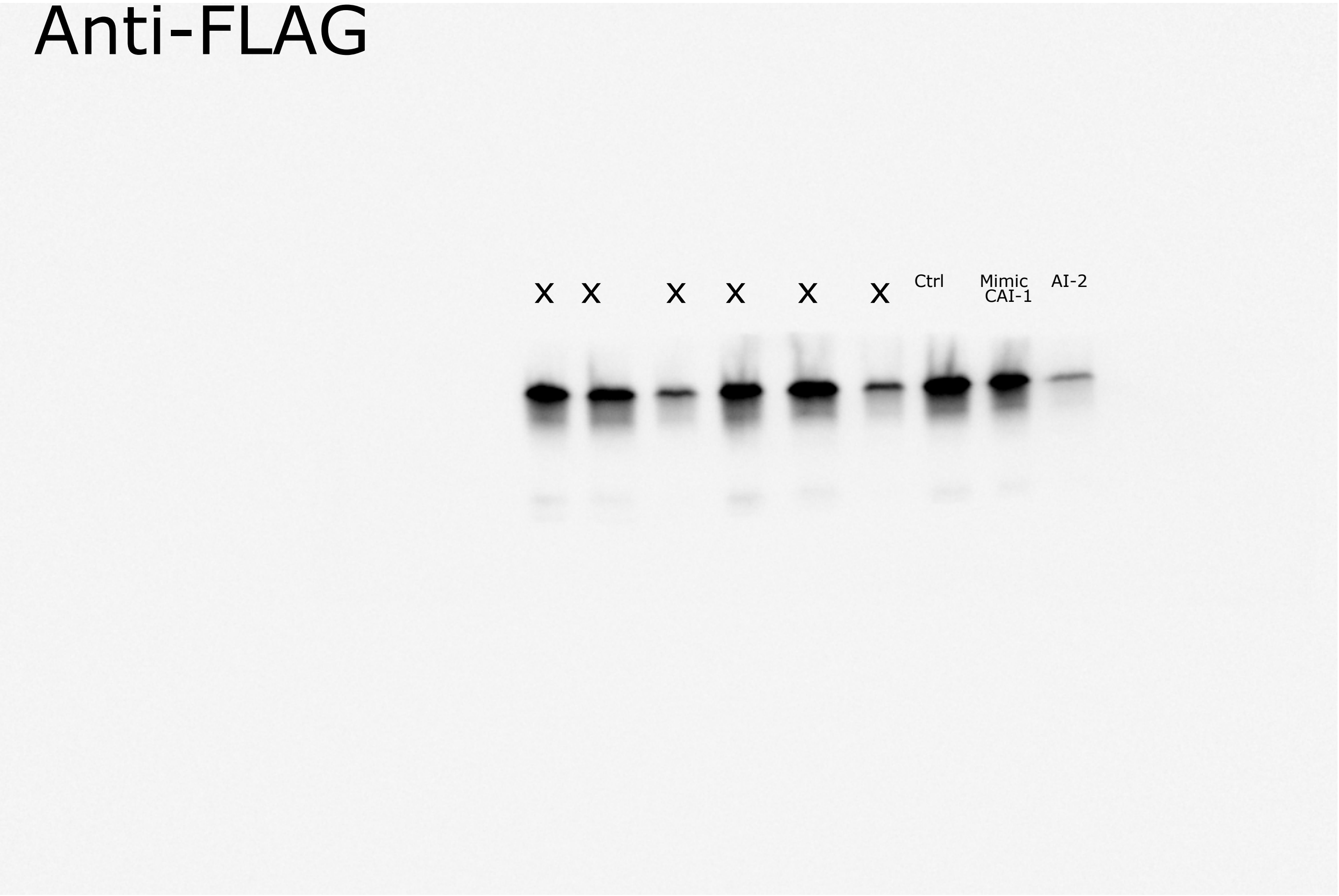

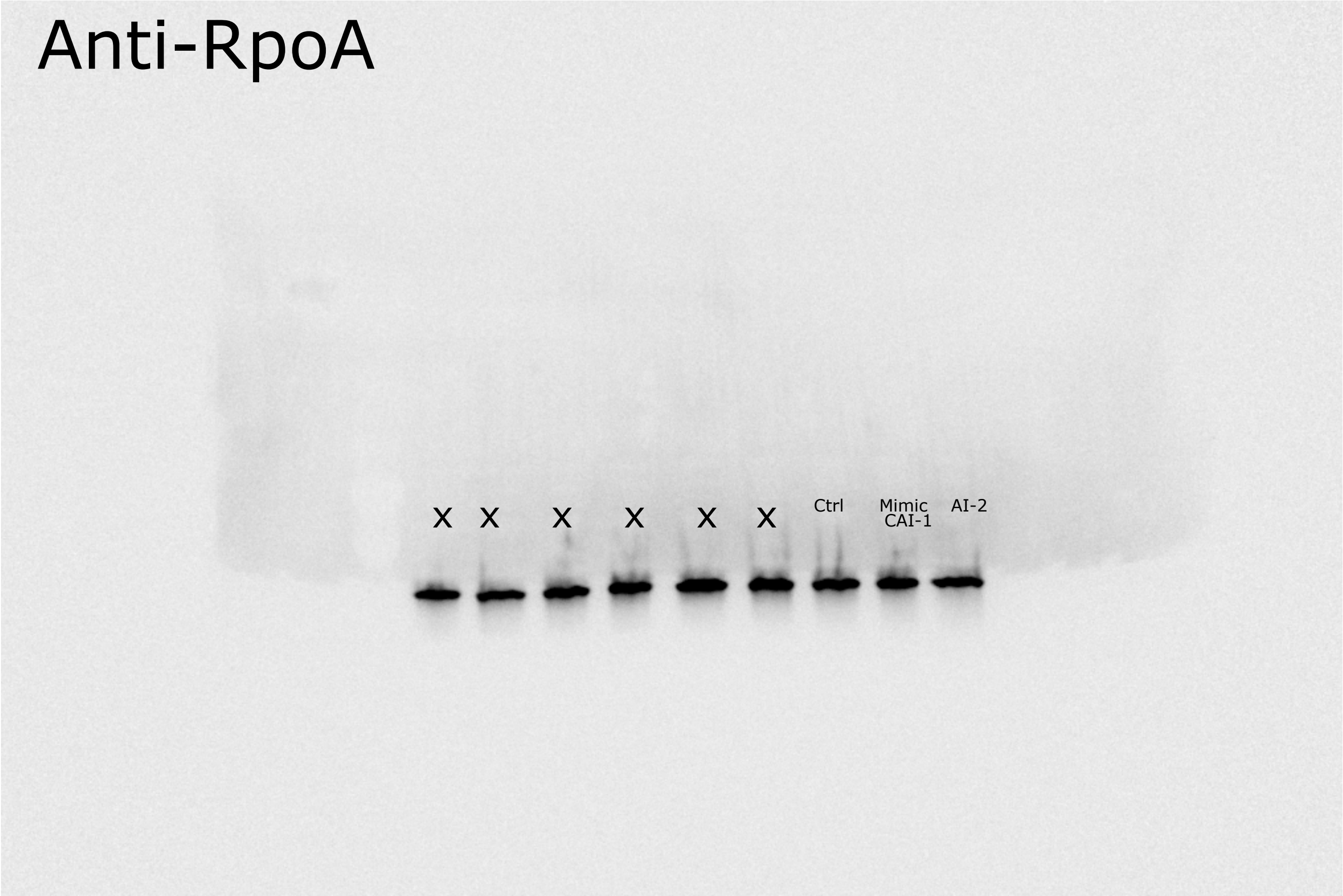

Figure 5E: anti-FLAG western blot

lane 1: OD600=0.05, CqsS^{S+R+}

lane 2: OD600=2.0, CqsS $^{S+R+}$

lane 3: OD600=0.05, CqsS $^{S-R+}$

lane 4: OD600=2.0, CqsS $^{S-R+}$

Figure 5B: anti-FLAG western blot

lane 1: OD600=0.05, CqsS^{S+R+}LuxPQ^{S+R+}

lane 2: OD600=2.0, CqsS^{S+R+}LuxPQ^{S+R+}

lane 3: OD600=0.05, SWAP

lane 4: OD600=2.0, SWAP

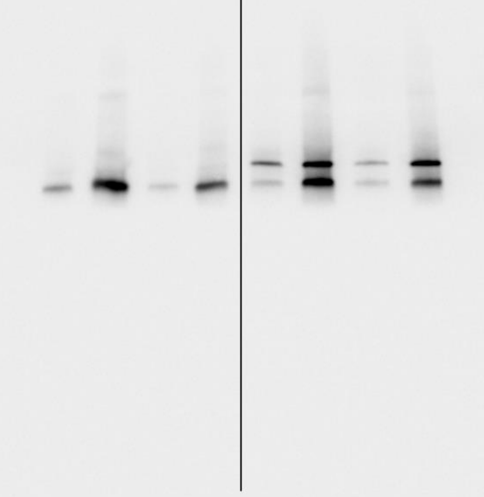

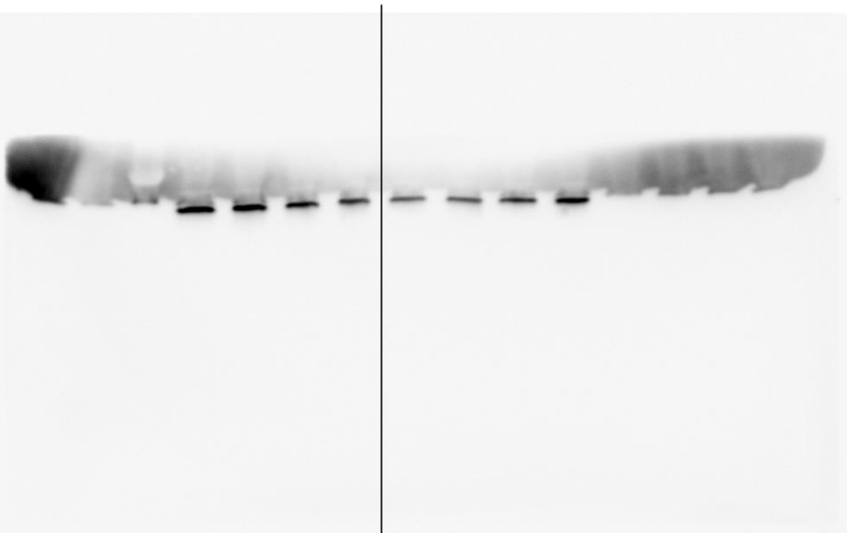

Figure 5E: anti-RpoA loading control

lane 1: OD600=0.05, CAI-1^{S+R+}

lane 2: OD600=2.0, CAI-1 $^{S+R+}$

lane 3: OD600=0.05, CAI-1 $^{S-R+}$

lane 4: OD600=2.0, CAI-1 $^{S-R+}$

Figure 5B: anti-RpoA loading control

lane 1: OD600=0.05, CAI-1^{S+R+}AI-2^{S+R+}

lane 2: OD600=2.0, CAI-1^{S+R+}AI-2^{S+R+}

lane 3: OD600=0.05, SWAP

lane 4: OD600=2.0, SWAP

Gel images for S6 Fig

Anti-FLAG

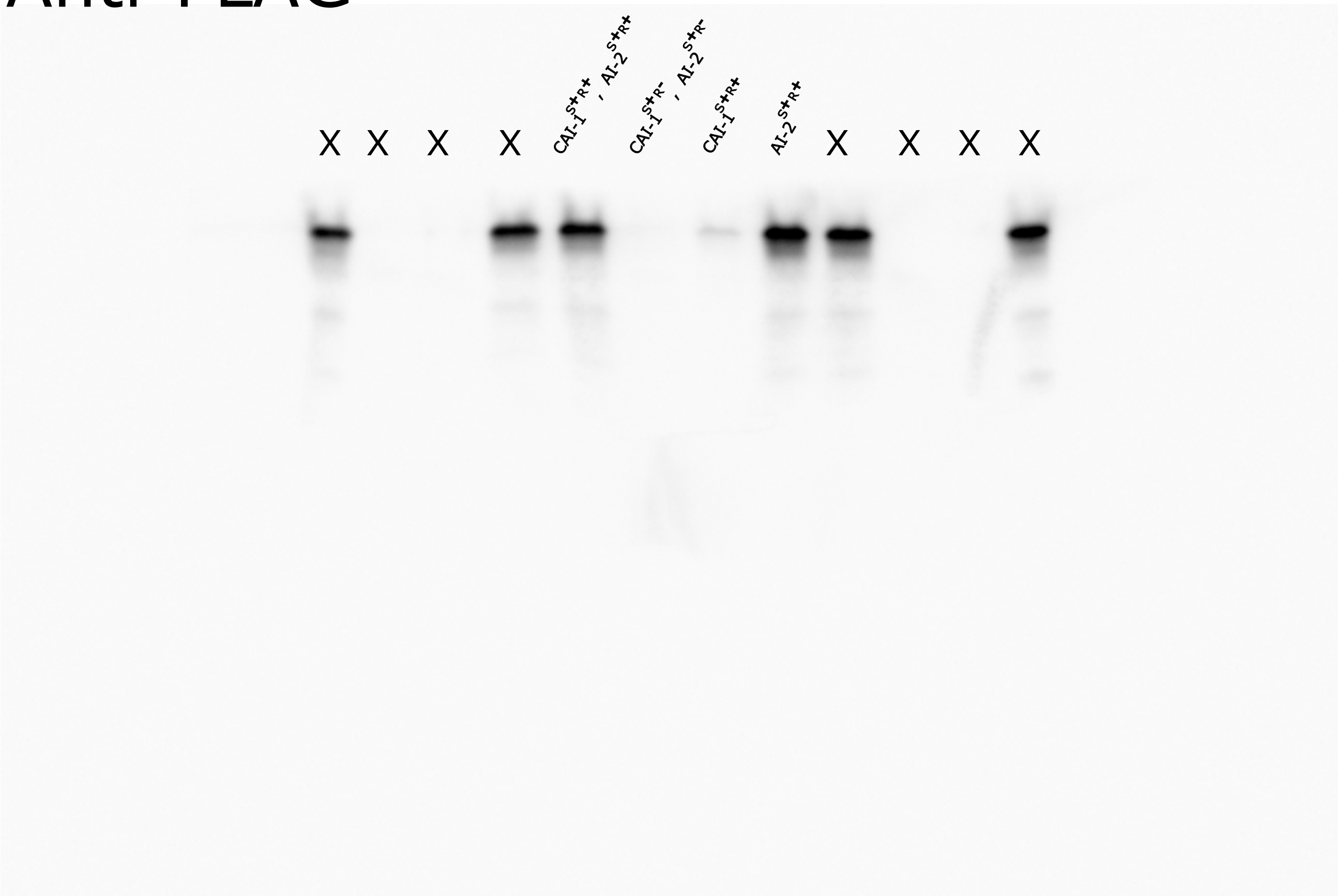

Anti-RpoA

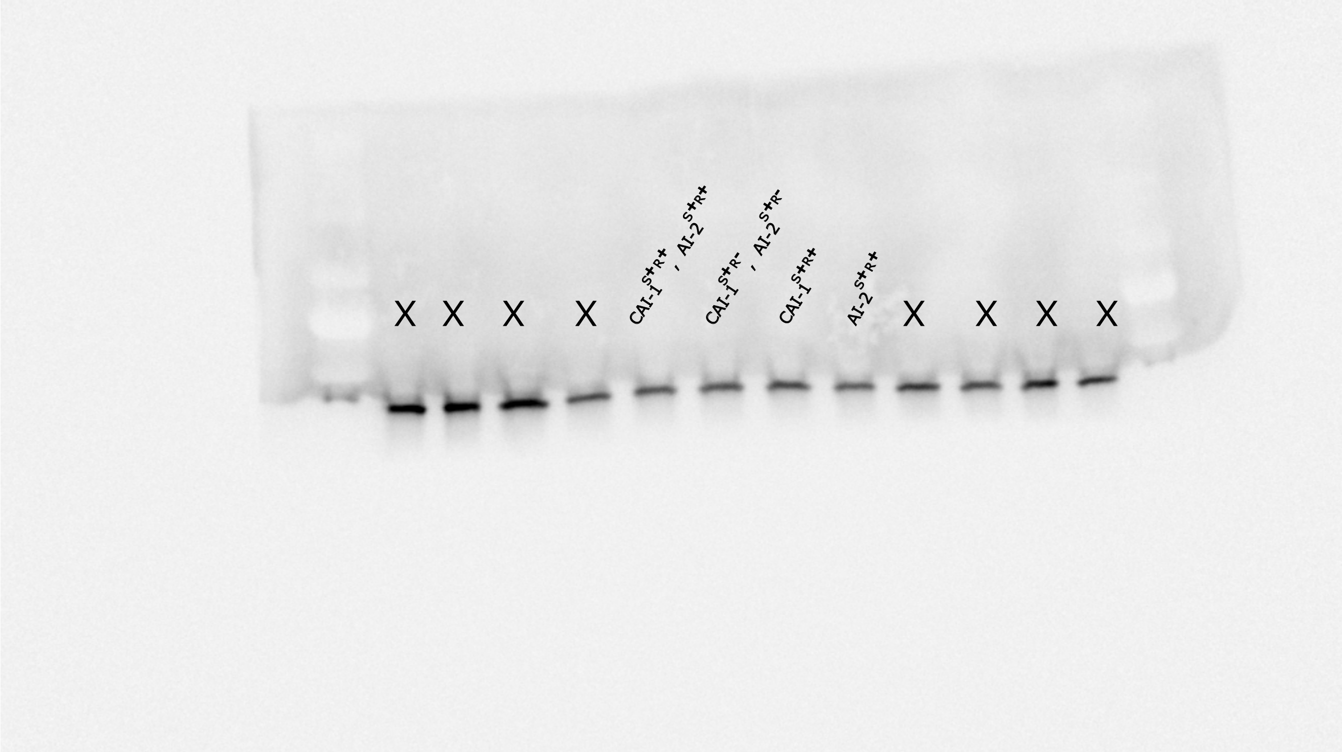

Supplement: S1 Raw Images — (PDF) [file pbio.3000429.s013.pdf]
