# Supplementary material for: The intragenus and interspecies quorum-sensing autoinducers exert distinct control over Vibrio cholerae biofilm formation and dispersal
Source: PLoS Biol. 2019 Nov 11;17(11):e3000429. doi: 10.1371/journal.pbio.3000429 (PMC6872173; doi:10.1371/journal.pbio.3000429)
Supplement: Attachment — Submitted filename: Bridges_2019_Response to Referees.docx [file pbio.3000429.s014.docx]

Dear Editor,

Thank you for handling our manuscript. We appreciate the referees’ comments and your guidance about how best to revise the paper. We are pleased that all four referees found the topics we covered to be of interest. The reviewers kindly provided us suggestions to improve our manuscript. We have taken these suggestions to heart and revised the manuscript exactly as requested. Most notably, we performed complementation experiments, quantified Western blots, discussed in more detail the inactivity of synthetic CAI-1 in biofilms, clarified our writing about self sensing, and included additional information regarding strain construction. Since the initial submission of this manuscript, we have extended our results to control of *Vibrio cholerae* virulence. We find that, consistent with our results regarding biofilm formation, relative to CAI-1, AI-2 plays the dominant role in controlling virulence factor production. We have included these new results in this resubmission. A point-by-point list of our responses to the referees’ comments is provided below. The referees’ comments are in black text and our responses are in blue text. We have also provided the revised text with all changes highlighted.

We hope you now find the manuscript acceptable for *PLOS Biology*. Please let us know if you need anything more.

Yours,

Bonnie Bassler

**Reviewer #1:**

The manuscript describes the interaction of two QS systems, CAI and AI-2 in V. cholerae to better define how these two systems can impinge on the same phosphorelay system, LuxO/U, to control phenotypic responses. The work largely focuses on a number of genetic constructs to determine these interactions. The authors conclude that CAI-1 is used to detect self and AI-2 to detect non-self and that the QS is largely driven by the AI-2 system.

The authors have clearly undertaken a considerable amount of work here to tease out these effects. One consequence is that the writing and text is very dense and hence it is hard to follow at times. There is a fair bit of narrative in the results, which if removed might help with the focus on interpreting the outcomes. Some of these are quite subjective, e.g. impervious, exquisite etc and are prone to hyperbole.

We thank Reviewer #1 for his/her comments. We regret that the referee found the text difficult to follow in places. The conundrum is that the other three referees all specifically noted that the manuscript is well written and straightforward to read/understand. In order to improve, we have gone through the text, particularly in the places Referee 1 pointed out, and tried to clarify our wording.

For example:

Previous version, we state (line 247):

“Together, our results exploring QS repression of biofilm formation and activation of light production show that WT *V. cholerae* is impervious to exogenously supplied CAI-1* but is exquisitely sensitive to exogenously supplied AI-2.”

Resubmitted manuscript, we state (line 267):

“Together, our results exploring QS repression of biofilm formation and virulence factor production and activation of light production show that WT *V. cholerae* shows little response to exogenously supplied Mimic^CAI-1^ but is highly sensitive to exogenously supplied AI-2.”

While it is appreciated that the authors used a different biofilm set up, this section could be omitted or considerably shortened to cut down on the text.

With respect, this section is core to the manuscript. In order for the reader to understand the new assay developed in this manuscript and put our findings into the context of previous biofilm measurements in *V. cholerae*, a description of our new approach is necessary. Furthermore, we anticipate that this new assay could be adopted by members of the field who want to study *V. cholerae* biofilm development and especially the under-studied dispersal portion of the lifecycle, which our new assay enables. A main goal of the manuscript is to report the new assay and how one performs it.

Similarly, given that the-self signalling experiments are convoluted and suggest there is no self signalling, that data can be omitted—although the final conclusions suggest that V. cholerae uses these as self sensing systems.

We are grateful for this comment, especially in light of comments about self sensing made by two of the three other referees. Our interpretation is that we were not clear in our writing concerning the difference between sensing the same or related bacterial species (which we refer to as “kin sensing” in the manuscript) and when an individual cell both secretes and detects its own autoinducer without the molecule being shared with the population (which we refer to as “self sensing” in the manuscript, and please see refs 40, 41). Indeed, as detailed below, referee #4 also had trouble understanding this distinction. In response, we have carefully rewritten the sections to be clear in every instance in which we mention each type of sensing. Furthermore, at the first use self sensing we include the following statement (line 383): “Recently, several bacterial QS circuits have been shown to be capable of self sensing, in which an individual cell releases and detects the autoinducer that it, itself, synthesized, without sharing this autoinducer with the community, by an autocrine-like mechanism (Fig 7A) [41,42]. For self sensing to occur, the cells must harbor sufficient levels of the receptor to capture/bind the released molecule prior to it diffusing away [43]. Importantly, self sensing is distinct from kin sensing via QS. Kin sensing occurs when bacteria of the same or closely related species share autoinducers among the cells in the vicinity.”

The authors argue throughout the manuscript that it is a density dependent phenomenon observed. However, the QS field seems to largely acknowledge that the accumulation of signals due to local signal concentration may also regulate these phenotypes. Since their system, and plankton cells are in a closed system, the signal concentration will track with cell number and hence it is not possible to differentiate the two. Additionally, batch systems will become limited in oxygen and nutrients due to metabolism, which also tracks with cell number and thus, it is thus possible that the batch biofilm system gives different results for any of these reasons.

Indeed. As the referee notes, autoinducer molecules build up locally and they do so in a cell density-dependent manner. That is the mechanism underlying quorum-sensing-controlled behaviors.

Given that they see the same changes in AphA and HapR in both planktonic and biofilm cells, it suggests this is not a biofilm specific phenotype.

We agree. Throughout the text, we carefully compare our results in this new biofilm assay to well established quorum-sensing readouts in planktonic cultures. With regard to AphA and HapR abundance, we make the same conclusion as the reviewer and that allows us to have confidence in the results from the new assay. Indeed, we wrote (line 178), “These results suggest that AphA and HapR levels vary inversely in response to changes in cell density, and that relationship is maintained in both biofilm and planktonic cells. Thus, the core behavior of the QS system is conserved in both growth modes.”

One of the biggest concerns is that the chemically synthesised CAI-1 does not induce the CAI-1 pathway for biofilms. In contrast a completely different chemical does. Thus, how can the authors justify that this system is responding to CAI-1? This has significant consequences for the rest of the data dependent on adding back the alternative chemical. It would also be important to not call the artificial compound CAI-1* as that is easily confused with the native signal CAI-1. A clearly different name should be used to help the reader. Why would CAI-1 form micelles for the biofilm culture but not the planktonic culture at the same concentrations?

We agree. We do not understand why synthetic CAI-1 does not activate the CqsS phosphatase activity in biofilms but it does in planktonic cells. In response to this comment, coupled with those from referees 2 and 3, we have expanded our discussion of this issue in the text. We provide more thoughtful suggestions for mechanisms that could account for the inactivity of the synthetic CAI-1.

Specifically, we state (line 523), “We were surprised that synthetic CAI-1, while active when provided to the CAI-1 reporter strain growing in the planktonic state, showed no activity when administered to growing biofilms (S2B Fig). One possibility is that the amphipathic character of CAI-1 prevents it from penetrating the biofilm matrix. A recent study suggests that endogenously-produced CAI-1 partitions into outer membrane vesicles, which stabilizes the molecule and facilitates its transmission between planktonic cells [46]. Thus, it is possible that synthetic CAI-1 partitions into vesicles or makes micelles in water, and becomes inaccessible to biofilm cells encapsulated in a matrix. This is clearly not the case for the polar AI-2 and Mimic^CAI‑1^ molecules that are active in both our planktonic and biofilms assays. While we do not know if these, or other mechanisms underlie the inactivity of exogenously-administered CAI-1 in our biofilm assays, using the CAI-1 surrogate, Mimic^CAI-1^, allowed us to overcome this experimental challenge to investigate how the activation state of each QS receptor controls biofilm formation and dispersal. Despite the inactivity of our exogenously-supplied synthetic CAI-1 in biofilms, our experiments (Fig 5) demonstrate that endogenously-produced CAI-1 is active within biofilms.”

Regarding the referee’s second good point here, it is certainly not our intention to confuse readers with the similar CAI-1 and CAI-1* naming conventions. Based on this referee’s recommendation, in the revised text, we call the synthetic molecule Mimic^CAI-1^. Also, in the revised text, we lay out our naming convention at the first use.

The Western blot data should be quantified as the differences are quite subtle for some. For example, it is difficult to judge from the image if CqsS is more abundant that LuxQ at high density.

Agreed. As suggested, in the revised text, we now include quantification of all Western blots, each from three biological replicates. As an example, in the blot in which we compare LuxQ and CqsS abundance (Fig 5B), we now include the quantified LuxQ/CqsS ratio in the revised figure and we provide that new figure here. The quantification confirms that, in WT cells, CqsS is more abundant than LuxQ at HCD. By contrast, when the receptor loci are exchanged, LuxQ is more abundant than CqsS at HCD.

New Fig 5B

How do the authors rationalise that CqsS is really only active at LCD, but that the protein is considerably more highly expressed at HCD, while there is only an apparently modest increase in LuxQ, despite it essentially being the dominant system here.

As detailed in the text, our data show that CqsS phosphatase is activated at surprisingly low cell densities because the receptor binds to CAI-1 under this condition. Moreover, the CqsS phosphatase remains active throughout continued growth, independent of receptor concentration. We never argue that CqsS is only active at LCD. The LuxPQ phosphatase, by contrast, is only activated by AI-2 at significantly higher cell densities. A main conclusion of this study is that there exists a large difference in the cell density thresholds at which the phosphatase of each receptor is activated. The coincidence detection feature of the system, rather than relative receptor levels, is what enables the LuxPQ kinase to dominate for the majority of the growth cycle. Furthermore, we note in the Discussion (lines 541-549) that, at very high cell densities, LuxPQ kinase activity can be overridden by CqsS phosphatase.

Why were experiments conducted at 30C in M9 salts medium, which has low NaCl compared to most work conducted with Vibrio species and has a complex nutrient and nitrogen source of glucose and casamino acids,? Perhaps this goes a long way to explaining the differences obtained here compared to the other studies, as noted in the discussion.

Unlike some other marine vibrios, the human pathogen *V. cholerae* grows robustly in media containing comparatively low salt concentrations, including LB, SOC, and M9. Thus, these three media have been adopted by the *V. cholerae* field. In this work, we are following the field convention by using SOC and M9. Specifically regarding M9, this minimal medium is routinely used for *V. cholerae* imaging analyses because of the substantially lower background fluorescence produced compared to LB or SOC. We include the following references as examples:

1.	Martinez RM, Megli CJ, Taylor RK. Growth and Laboratory Maintenance of Vibrio cholerae. Curr Protoc Microbiol. 2010;0 6: Unit-6A.1. doi:10.1002/9780471729259.mc06a01s17

2.	Miller MB, Skorupski K, Lenz DH, Taylor RK, Bassler BL. Parallel quorum sensing systems converge to regulate virulence in Vibrio cholerae. Cell. 2002;110: 303–314. doi:10.1016/s0092-8674(02)00829-2

3.	Fong JC, Rogers A, Michael AK, Parsley NC, Cornell W-C, Lin Y-C, et al. Structural dynamics of RbmA governs plasticity of Vibrio cholerae biofilms. Newman DK, editor. eLife. 2017;6: e26163. doi:10.7554/eLife.26163

4.	Yan J, Sharo AG, Stone HA, Wingreen NS, Bassler BL. Vibrio cholerae biofilm growth program and architecture revealed by single-cell live imaging. PNAS. 2016;113: E5337–E5343. doi:10.1073/pnas.1611494113

The AI-2 is resented as a ‘universal’ signal and this does not seem to really be the case. For the Vibrio spp, E. coli and Salmonella, this seems correct, but in many other species, it has been shown to have no true signalling function, or at least none that have yet been determined. Similarly, biofilm formation is QS controlled for some, but not all bacteria and these points should be amended for accuracy.

Genomic studies indicate that many bacterial species do not encode obvious AI-2 receptors, yet thousands of bacterial species possess the AI-2 synthase LuxS, and in every case tested, they make AI-2. In most cases, whether or not AI-2 plays a quorum-sensing role has not been studied. To our knowledge, AI-2 is the most broadly used autoinducer discovered to date. Nonetheless, we take the referee’s caution to heart. In response, we have removed the word “universal” and instead used terms like “broadly made” or “widely produced”. We do note that in the original submission and in this revised submission, we have been careful to restrict our conclusions regarding AI-2 detection exclusively to *V. cholerae*.

Regarding the second point in this passage, we agree that quorum sensing does not control biofilm formation and dispersal in all bacteria. Our introduction reads (line 31), “Frequently, QS controls the development of biofilms, which are surface-associated communities of bacteria that secrete an adhesive extracellular matrix [3,4]. “

Were the signals added from time 0 of the experiments?

The autoinducers were added immediately after cells were allowed to attach to glass coverslips. Our procedure is/was described in the Methods. The text states (line 627), “In all cases, autoinducers were added to cells post-attachment to glass coverslips, immediately after the final washing step described above.”

One suggestion might be that the integration of CAI-1 and AI-2 is one of biochemistry, dependent on signal-receptor binding constants, receptor numbers, as well as kinase/phosphatase activity rates. thus, it may be cleaner to compare these for purified components if possible given all of the complexities of the various interacting regulators, differences in biofilm/planktonic responses for the artificial vs real CAI signal etc. Clearly, not in the scope of this manuscript though.

Yes indeed! We would love to do this experiment. Unfortunately, despite nearly two decades of effort, no one has yet successfully purified the CqsS and LuxPQ transmembrane bound receptors. Thus, the field and we are limited to indirect readouts of kinase/phosphatase activity.

**Reviewer #2:**

The study by Bridges and Bassler focuses on the role of quorum sensing (QS) for biofilm formation and dispersal of the bacterial pathogen Vibrio cholerae. Specifically, the authors analyze two autoinducer molecules, CAI-1 and AI-2, which signal through distinct membrane-bound receptors (sensors) to the same response regulator, LuxO. To this end, the authors establish a live-cell biofilm assay, and show that the QS system functions as a co-incidence detector, since both autoinducers need to be present simultaneously to trigger phosphate flow reversion (sensor kinase to phosphatase) and repression of biofilm formation. Intriguingly, CAI-1-signaling indeed occurs by QS not by “self sensing”, and the system responds with much greater sensitivity to CAI-1 than to AI-2, suggesting that the detection of kin is a prerequisite for the detection of and response to non-kin bacteria.

In summary, this interesting and thorough study addresses and significantly contributes to answering the longstanding question of how different signaling pathways, which coincide on a common response regulator, contribute to control complex bacterial behavior like biofilm formation or virulence. Based on the concepts of coincidence detection and vastly different thresholds at which CAI-1 and AI-2 trigger signaling, it appears that V. cholerae engages on biofilm dispersal when an area gets “overcrowded” by non-kin bacteria and only forms a new biofilm in an unoccupied area.

The study is well controlled, and the story unravels in a straightforward manner. A few rather minor points should be addressed to further improve the manuscript.

We thank Reviewer #2 for his/her positive comments.

Specific points

1) Fig. 2BD: The impressive biofilm phenotypes of the V. cholerae mutant strains lacking vpsL or hapR should be complemented by providing the corresponding genes on a plasmid.

Yes. these are important controls to include. As recommended, we conducted complementation experiments with the Δ*hapR* and Δ*vpsL* mutants presented in Fig 1. To avoid including antibiotics in the assays, we integrated a single copy of each gene into the genome at an ectopic locus, *lacZ*, a strategy we and others routinely use. Complementation to the wildtype phenotype occurred in both cases. These results are now described in the text and the data are reported in the new S1 Fig.

New S1 Fig

2) Fig. 3BD: For the mRuby3 fluorescence, standard deviation (shading) is shown but not the mean data points. The corresponding data points and lines should be added.

We added the data as requested. The corrected plots are shown here and they now make the new Fig 3B,D of the revised manuscript.

New Fig 3 B,D

3) Fig. 5E: The “green circles” are actually black circles on a green line. Please reconcile.

As requested, we have corrected the color key and the figure caption for Fig 5E. The revised figure is provided below. The text of the caption now states, “ (E) Middle panel: Quantitation of biofilm biomass over time for the *V. cholerae* CAI-1^S+R+^, AI-2^S+R+­­^ (blue circles, blue line) and CAI-1 ^S-R+^ (open circles, green line) strains.”

New Fig 5E

4) Fig. S1A: Bright field projections of V. cholerae biofilms (as in Fig. 4A) would be informative.

As requested, in the revised submission, we include brightfield images for this figure (now S2 Fig) and for S3 Fig. The new figures are shown here.

New S2A Fig

New S3A Fig

5) Unexpectedly, synthetic CAI-1 is inactive under the live-cell biofilm formation conditions used in the study, while active under planktonic conditions in shaken culture. The possible reason given by the authors – “micelle formation” (l.203) – does not seem to be overtly plausible, since in both cases the same medium was used (and hence, micelle formation of CAI-1 is likely similar). Also, the results are surprising in light of the finding that CAI-1-mediated signaling is indeed QS not “self-sensing” (Fig. 7). These observations should be more thoroughly outlined in the Discussion section.

As mentioned in our response to Referee #1, here for Referee #2, and below for Referee #3, we do not understand why synthetic CAI-1 does not activate the CqsS phosphatase in biofilms but it does in planktonic cells. We have expanded our discussion of this issue in the text. We provide more thoughtful suggestions for mechanisms that could account for the inactivity of the synthetic CAI-1.

Specifically, we state (line 523), “We were surprised that synthetic CAI-1, while active when provided to the CAI-1 reporter strain growing in the planktonic state, showed no activity when administered to growing biofilms (S2B Fig). One possibility is that the amphipathic character of CAI-1 prevents it from penetrating the biofilm matrix. A recent study suggests that endogenously-produced CAI-1 partitions into outer membrane vesicles, which stabilizes the molecule and facilitates its transmission between planktonic cells [46]. Thus, it is possible that synthetic CAI-1 partitions into vesicles or makes micelles in water, and becomes inaccessible to biofilm cells encapsulated in a matrix. This is clearly not the case for the polar AI-2 and Mimic^CAI‑1^ molecules that are active in both our planktonic and biofilms assays. While we do not know if these, or other mechanisms underlie the inactivity of exogenously-administered CAI-1 in our biofilm assays, using the CAI-1 surrogate, Mimic^CAI-1^, allowed us to overcome this experimental challenge to investigate how the activation state of each QS receptor controls biofilm formation and dispersal. Despite the inactivity of our exogenously-supplied synthetic CAI-1 in biofilms, our experiments (Fig 5) demonstrate that endogenously-produced CAI-1 is active within biofilms.”

6) The authors address a possible dose-response relationship of sensor kinase (CqsS, LuxQ) production and QS in the course of biofilm formation and dispersal (Fig. 5). The observations could also be discussed in the context of Vibrio QS autoinducers acting dose-dependently as “timers” – at least under planktonic conditions (Anetzberger et al., 2012, PLoS One 10: e48310).

We thank the reviewer for this insightful point that we failed to discuss in the original submission. We have included the following text in the revised Discussion section (line 472). “Thus, AI-2 accumulation is the limiting step for the transition from the LCD to HCD QS mode, and for driving the transition from biofilm growth to biofilm dispersal. Indeed, it is likely that the temporal offset in the accumulation of the two autoinducers is responsible for the observed asymmetry in biofilm control. Precedence for autoinducer accumulation asymmetry exists in the closely related organism, *Vibrio harveyi*, under planktonic growth conditions [44].”

7) l.306: … was similar in the … (not “identical”)

We have changed “identical” to “similar” in the revised document (now line 332).

**Reviewer #3:**

Vibrio cholerae controls major processes such as biofilm formation and virulence via cell-cell communication. Several systems are involved in this regulation, in particular the CAI-1-dependent and AI-2-dependent systems. CAI-1 and AI-2 are autoinducers used by V. cholerae to measure its own abundance and the abundance of total bacterial cells, respectively. The two autoinducers bind distinct receptors but the signal is relayed to the final effectors through the same pathway. This layout has made it difficult to understand the benefits of each part of the system.

The authors sought to examine the contribution of each autoinducer to the transmission of the information and thus to the control of V. cholerae behavior. For that purpose, they developed a live-cell biofilm formation and dispersal assay.

This paper asks a very interesting question of great importance to understand the biology of V. cholerae and its behavior during its life-cycle.

The paper presents elegant experiments, is well-written, well organized and easy to read.

We thank Reviewer #3 for these positive comments.

However, I have a few comments that are detailed below.

MAJOR COMMENTS

1. Fig. 7 and lines 364 to 366: This is a nice experiment. However, its presentation is a bit too "black-and-white". The authors write that "if self sensing occurs, in co-culture, the autoinducer made by the secrete-and-sense strain would trigger its HCD mode, while the sense-only strain would remain in LCD mode". I think this statement (+ Fig. 7B) should be toned down by adding that another possibility would be that the sense-only cells could also display a delayed response compared to the secrete-and-sense cells. Also, is the difference that we see between the green and black lines in Fig. 7D not significant? Couldn't that reflect a difference in response between the 2 strains? If so, scenario 1 is to be put forward.

We thank the reviewer for this idea that improves the quality of the manuscript. Although the difference in the green and black lines in Fig 7D is modest (below, note the log scale), it is significant, as the reviewer points out. Therefore, we now include the intermediate state in which a small amount of self sensing does occur but the CAI-1 autoinducer is largely shared with the community. We also provide a revised Fig 7B that shows this case (see below). We have toned down the language in this paragraph. We can nonetheless continue to conclude that QS, not self sensing, is the major driving force behind activation of this circuit.

New Fig 7

Besides that, considering that the interest of the authors is to examine the role of the autoinducers in biofilm formation, it would be very informative to perform the same experiment in biofilm-forming conditions. This would factor in the fact that the autoinducers probably display a different availability than in shaken cultures.

Indeed, this is a fascinating area for exploration. However, isolating cells from within biofilms for flow cytometry-based analysis has not been successful. Therefore, we believe this experiment is beyond the scope of the current manuscript.

2. Great efforts have been made for the description of the experiments in the materials and methods section. However, I did not see any precise description of strain construction. It is necessary to add that information, preferentially to the supplementary material.

We thank the reviewer for this comment, and we regret that our strain construction methods were insufficient. In the updated version of the manuscript, we have included the identities of the parent for each strain produced in this work and we have provided a table with the primers used for constructions. We hope that this information, combined with the companion text in the Methods section that fully describes our strain construction protocol and provides references, is sufficient for publication.

MINOR COMMENTS

1. Line 198: Please explain in one sentence how the heterologous readout for HapR-controlled QS activity works (which promoter is used for example).

We have made the suggested change to the revised manuscript. The text now says (line 203), We confirmed that our synthetic CAI-1 is fully active in this reporter strain by monitoring bioluminescence emission from a chromosomally integrated luciferase (*luxCDABE*) operon driven by the QS-controlled native promoter. This reporter is routinely-used as a heterologous readout for HapR-controlled QS activity in *V. cholerae* [28].”

2. Lines 201 to 204: The authors state that synthetic CAI-1 is inactive in biofilm conditions probably because of micelle formation. Please briefly explain why (why would there be micelle formation and why these micelles would perturb the functioning of CAI-1 and not CAI-1*). Furthermore, on Fig. S1, although CAI-1 and CAI-1* both activate the production of light, the response profiles are different. Indeed, CAI-1* induces a very sharp response compared to CA1-1. Although this does probably not explain the disparity in biofilm formation, this difference in activity would be worth mentioning in the text.

As mentioned in our responses to Referees #1 and #2, we do not understand why synthetic CAI-1 does not activate the CqsS phosphatase in biofilms but it does in planktonic cells. We have expanded our discussion of this issue in the text. We provide more thoughtful suggestions for mechanisms that could account for the inactivity of the synthetic CAI-1.

Specifically, we state (line 523), “We were surprised that synthetic CAI-1, while active when provided to the CAI-1 reporter strain growing in the planktonic state, showed no activity when administered to growing biofilms (S2B Fig). One possibility is that the amphipathic character of CAI-1 prevents it from penetrating the biofilm matrix. A recent study suggests that endogenously-produced CAI-1 partitions into outer membrane vesicles, which stabilizes the molecule and facilitates its transmission between planktonic cells [46]. Thus, it is possible that synthetic CAI-1 partitions into vesicles or makes micelles in water, and becomes inaccessible to biofilm cells encapsulated in a matrix. This is clearly not the case for the polar AI-2 and Mimic^CAI‑1^ molecules that are active in both our planktonic and biofilms assays. While we do not know if these, or other mechanisms underlie the inactivity of exogenously-administered CAI-1 in our biofilm assays, using the CAI-1 surrogate, Mimic^CAI-1^, allowed us to overcome this experimental challenge to investigate how the activation state of each QS receptor controls biofilm formation and dispersal. Despite the inactivity of our exogenously-supplied synthetic CAI-1 in biofilms, our experiments (Fig 5) demonstrate that endogenously-produced CAI-1 is active within biofilms.”

Additionally, as suggested, in the revised Results, we discuss the differences in timing of activation of the *lux* reporter among CAI-1 and Mimic^CAI-1^ treatment (line 206): “When grown in shaken, planktonic conditions, both CAI-1 and Mimic^CAI-1^ induced an ~1000-fold increase in light production by the CAI-1 reporter strain, although Mimic^CAI-1^ activated the reporter earlier, at a lower cell density.”

3. Please indicate in the materials and methods section (microscopy paragraph) why the biofilm data are normalized to the peak biofilm biomass for the control strain.

As suggested, we have included the following statement in the revised manuscript (line 650): “In all plots, data were normalized to the reference strain/conditions for that day rather than as absolute biofilm biomass values due to slight variability in the amount of biofilm formation (across all strains) that occurred from day to day.”

4. Fig. 5E middle panel and line 308: Albeit in a delayed manner, the biofilm still disintegrates. Please specify what signal could induce the dispersal.

We agree. Our thought is that the mutant that lacks *cqsA* locks the CqsS receptor into kinase mode, conferring the same quorum-sensing low-cell-density phenotype as that exhibited by the *luxO D61E* mutant and not that of the more extreme Δ*hapR* mutant presented in Fig 1. We refer the referee to our discussion of the different phenotypes of the mutants in Fig 1:

We state, “Notably, the phenotype of the Δ*hapR* strain was more extreme in its preference for the biofilm state than that of the *luxO D61E* strain, consistent with the downstream position and direct function of HapR in regulation of biofilm formation. Specifically, LuxO D61E drives constitutive production of the Qrr sRNAs (Fig 1A) [36]. The Qrr sRNAs activate translation of AphA and repress translation of HapR, and they positively and negatively regulate other targets [37,38]. Thus, in the *luxO D61E* strain, unlike in the Δ*hapR* strain, some HapR is present that can activate modest biofilm dispersal, and, moreover, other Qrr-regulated targets also promote biofilm dispersal in the LuxO D61E mutant. These features of the QS circuit have been reported previously and underlie the difference in phenotypes between the two mutants [37].”

5. Fig. 6: I wish the authors had also presented biofilm assays. Although lower than that of the wt, the patterns of light production are different in the red and green strain and it would have been interesting to see if this would translate to a different biofilm formation/dispersal behavior.

We appreciate this good idea. In response, have now included these results in new S7 Fig, provided below. Both mutants lacking the gene encoding a single autoinducer synthase display modest dispersal defects (similar to that of the *luxOD61E* mutant in Fig. 1).

New S7 Fig

6. The authors perform their experiments in strain V. cholerae O1 El Tor which harbors the pathway leading to biofilm formation and virulence as described in Fig. 1. Other strains with a Classical background do not possess HapR. Could the authors discuss their results in light of this consideration?

The referee is correct, and this is a feature that many *V. cholerae* experts are curious about. In the revised Discussion, we have included text regarding the differences between strains and their quorum-sensing components. We write (line 550): “Here, we focused exclusively on *V. cholerae* El Tor biotype strain C6706, which possesses a functional QS system. Some *V. cholerae* strains, of both the El Tor and Classical biotypes, harbor mutations in *hapR* that render the HapR proteins nonfunctional [47]. Thus, in these strains, HapR-directed QS control of biofilm behavior does not occur. Notably, an alternative signal transduction system, called VieSAB, can also be involved in controlling biofilm phenotypes via modulation of the levels of the second messenger cyclic diguanylate, particularly in the Classical biotype [48–50]. In *V. cholerae* strains possessing functional HapR, *vieSAB* is repressed by HapR at HCD, so we propose that, in these strains, QS controls biofilm dispersal. We speculate that in *V. cholerae* strains lacking a functional HapR, biofilm dispersal could be controlled by the VieSAB pathway. Moreover, given the reduced propensity for *V. cholerae* *hapR* mutants to disperse from biofilms (Fig 2D), we further speculate that such mutants primarily occupy niches in which it is advantageous for *V. cholerae* to remain in the biofilm state for long periods, possibly including under HCD conditions.”

**Reviewer #4:**

I have read “The intra-genus and inter-species quorum-sensing autoinducers exert distinct control over Vibrio cholerae biofilm formation and dispersal” by Bridges and Bassler. The paper increases our understanding of the wiring of the well-studied quorum sensing system of biofilm formation and dispersal in V. cholerae. The paper presents elegant real time microscopy methods to detect transition from biofilm growth to dispersal. The paper is well written, and the data are presented clearly and convincingly. I recommend publication but suggest the authors consider additional evolutionary interpretations of the CAI-1 and AI-2 coincidence detector mechanism.

The most intriguing finding is that the two studied QS pathways, CAI-1 and AI-2, differ so much with respect to the densities at which they switch from biofilm promoting to dispersal inducing. I am convinced by this difference. QS signals that are not unique to a genotype have been discussed at length in the evolutionary theory literature: how is honest signaling possible in face of potential signal cheats and what is it that QS is measuring? Here, the authors propose that the two signals act as a coincidence detector: only when both signals are present will dispersal from the biofilm be induced. The authors interpret this evolutionarily, too: when the inter-species signal AI-2 is present due to non-kins present in a habitat, a small nascent community of V. c. would rapidly disperse due to high sensitivity to the “private” CAI-1 signal; this is interpreted as a mechanism to disperse and find more suitable habitats. Additionally, I think this data---quite beautifully---corroborates theory that predicts QS may evolve robustly as a means to infer kinship. Because bacteria grow in clonal patches in biofilms, therefore, a brief waiting period “after landing” is sufficient to allow for the evolution of more complex cooperative phenotypes that positively feedback and increase the genotypes fitness (Schluter…Foster & Mitri, 2016 PLoS Comput Biol). The CAI-1/AI-2 pathway may be first empirical evidence for this logic, with AI-2 acting as an additional measure for crowdedness that is presumably inversely related to the residual capacity of the habitat.

We thank Reviewer #4 for these very positive comments. In addition to changing the manuscript according to the reviewer’s minor comments (addressed below), as suggested, we have included the following evolutionary interpretation in the Discussion (line 488): “Thus, we propose that the CAI-1/CqsS arm serves as a filter that prevents the transition to HCD mode when fewer than the critical threshold number of kin cells are present, even in scenarios in which dense populations of non-kin bacteria are present (as judged by AI-2 levels). The activity we observe for the CAI-1/CqsS circuit is consistent with theoretical work suggesting that a possible evolutionary benefit of QS is that it enables bacteria to verify the presence of related neighbors prior to committing to potentially costly group behaviors, thereby limiting benefits to “cheaters” in the community [45]. We contrast the behavior of the CAI-1/CqsS circuit to that of the AI-2/LuxPQ circuit, which has a high cell-density dependent activation threshold. Thus, for *V. cholerae*, the buildup of AI-2 is the rate limiting step for satisfying the coincidence detector constraint.”

I recommend publication and append some minor comments that may improve the manuscript.

The article is rather long. The section (line 385 ff) on self-sensing vs QS seems a bit unnecessary. If I understand correctly, these are results from shaken cultures. In biofilms, however, local kinship can be inferred due to clonal growth as relatedness increases naturally over time in such spatially structured situations. Here, therefore, the vastly different sensitivity thresholds to the two signals may hold the key to sensing self from non-self.

With respect, we believe it is critical to keep this section in the manuscript. While we agree with the reviewer that local kinship arises due to the spatial structure of the biofilm, our intent with this section was to address a different, fundamental question: Does the same bacterial cell secrete and detect its own CAI-1 autoinducer or are autoinducers shared between cells. We acknowledge that, in a biofilm, CAI-1 may never leave the regional community.

Figure 7B is poorly explained and the legend refers to the main text for details (of which there are not many). A better legend (e.g. what are the shadings of the cells?) would improve the figure.

We thank the reviewer for this comment. In response, we have expanded the legend to Fig 7B. Also, in the revised Fig 7B, we added an intermediate state in response to a suggestion by Reviewer #3. The new figure is shown for reference. Regarding the legend, we now write, “(B) Predicted HCD gene expression level (shades of blue) over increasing cell density for co-cultured secrete-and-sense and sense-only strains if a circuit exhibits exclusive self-sensing behavior (top), QS behavior (middle), or an intermediate state in which both self sensing and QS occur (bottom).”

New Fig 7B

In addition, in response to the Reviewer’s suggestion, we have edited the main text to clarify this section. We did not intend to convey “kin” and “self” as synonyms as may have been the referee’s interpretation. Rather, we use “kin” to mean the same or related bacterial species, and we use “self” to mean when an individual cell both secretes and detects its own autoinducer without the molecule being shared with the population (please see refs 40, 41). As described in response to referee #1, we have now carefully defined these concepts in the revised text.

Figure 3: Was the AphA tagged experiment also started from an HCD overnight culture? Would we not expect, therefore, AphA to be low in the first hours, then high, and then decline (as is indeed the case for ahpa:hapr)?

The reviewer is correct. In the overnight cultures, AphA cannot be detected. In the two hours between dilution and the initiation of our time-lapse imaging procedure, AphA production commences and allows us to detect the protein. To clarify this issue, we have added the following text to the Results section (line 164), “The AphA-mNG signal increased following initiation of the biofilm assay, an increase that occurred prior to the start of image acquisition, and subsequently declined 4-fold over the lifetime of the biofilm relative to the constitutive reporter (Fig 3A, B).”

Can you comment on the observed or estimated diffusion rates of the two signals?

Unfortunately, the diffusion rates for CAI-1 and AI-2 have never successfully been measured. For this reason, we hesitate to speculate. However, we have added some text to the revised Discussion about the predicted properties of CAI-1. We state (line 523), “We were surprised that synthetic CAI-1, while active when provided to the CAI-1 reporter strain growing in the planktonic state, showed no activity when administered to growing biofilms (S2B Fig). One possibility is that the amphipathic character of CAI-1 prevents it from penetrating the biofilm matrix. A recent study suggests that endogenously-produced CAI-1 partitions into outer membrane vesicles, which stabilizes the molecule and facilitates its transmission between planktonic cells [46]. Thus, it is possible that synthetic CAI-1 partitions into vesicles or makes micelles in water, and becomes inaccessible to biofilm cells encapsulated in a matrix. This is clearly not the case for the polar AI-2 and Mimic^CAI‑1^ molecules that are active in both our planktonic and biofilms assays. While we do not know if these, or other mechanisms underlie the inactivity of exogenously-administered CAI-1 in our biofilm assays, using the CAI-1 surrogate, Mimic^CAI-1^, allowed us to overcome this experimental challenge to investigate how the activation state of each QS receptor controls biofilm formation and dispersal. Despite the inactivity of our exogenously-supplied synthetic CAI-1 in biofilms, our experiments (Fig 5) demonstrate that endogenously-produced CAI-1 is active within biofilms.”
